# TAT-PEP Alleviated Cognitive Impairment by Alleviating Neuronal Mitochondria Damage and Apoptosis After Cerebral Ischemic Reperfusion Injury

**DOI:** 10.1007/s12035-023-03404-w

**Published:** 2023-06-19

**Authors:** Pin Zhao, Jiapo Zhang, JianKe Kuai, Liya Li, Xuying Li, Namin Feng, Hailiang Du, Chen Li, Qiang Wang, Bin Deng

**Affiliations:** 1 Department of Anesthesiology & Center for Brain Science, The First Affiliated Hospital of Xi’an Jiaotong University, Xi’an, 710061 Shaanxi China; 2 Department of Anesthesiology, Xi’an No.3 Hospital, The Affiliated Hospital of Northwest University, Xi’an, Shaanxi 710018 People’s Republic of China; 3 Department of Emergency Medicine, School of Medicine, Xiang’an Hospital of Xiamen University, Xiamen University, Xiamen, 361102, Fujian China; 4 Department of Anesthesiology, The Second Affiliated Hospital of Dalian Medical University, Dalian, 116027, Liaoning China

**Keywords:** TAT-PEP, Cerebral ischemic reperfusion injury, Cognitive impairment, Neuronal mitochondria

## Abstract

Paired immunoglobulin-like receptor B (PirB) was identified as a myelin-associated inhibitory protein (MAIP) receptor that plays a critical role in axonal regeneration, synaptic plasticity and neuronal survival after stroke. In our previous study, a transactivator of transcription-PirB extracellular peptide (TAT-PEP) was generated that can block the interactions between MAIs and PirB. We found that TAT-PEP treatment improved axonal regeneration, CST projection and long-term neurobehavioural recovery after stroke through its effects on PirB-mediated downstream signalling. However, the effect of TAT-PEP on the recovery of cognitive function and the survival of neurons also needs to be investigated. In this study, we investigated whether pirb RNAi could alleviate neuronal injury by inhibiting the expression of PirB following exposure to oxygen–glucose deprivation (OGD) in vitro. In addition, TAT-PEP treatment attenuated the volume of the brain infarct and promoted the recovery of neurobehavioural function and cognitive function. This study also found that TAT-PEP exerts neuroprotection by reducing neuronal degeneration and apoptosis after ischemia–reperfusion injury. In addition, TAT-PEP improved neuron survival and reduced lactate dehydrogenase (LDH) release in vitro. Results also showed that TAT-PEP reduced malondialdehyde (MDA) levels, increased superoxide dismutase (SOD) activity and reduced reactive oxygen species (ROS) accumulation in OGD-injured neurons. The possible mechanism was that TAT-PEP could contribute to the damage of neuronal mitochondria and affect the expression of cleaved caspase 3, Bax and Bcl-2. Our results suggest that PirB overexpression in neurons after ischaemic-reperfusion injury induces neuronal mitochondrial damage, oxidative stress and apoptosis. This study also suggests that TAT-PEP may be a potent neuroprotectant with therapeutic potential for stroke by reducing neuronal oxidative stress, mitochondrial damage, degeneration and apoptosis in ischemic stroke.

## Introduction

Ischemic stroke is a primary circulatory and cerebral vascular disease that is associated with significant rates of morbidity, mortality and disability [1]. In particular, approximately 60–80% of survivors have permanent neurological deficits, such as cognitive impairment, sensory and motor deficits, which are a major burden on society and families [2]. However, the mechanism of ischaemic stroke is not well understood, and treatment is limited. How to promote recovery of neural function and reduce the rate of disability is therefore a pressing issue. It is also a challenging global problem.

The pathophysiological process of ischemic stroke is relatively complex. Among them, cerebral ischemia–reperfusion injury can lead to cerebral ischemia and hypoxia, causing damage to the structure and function of brain tissue, including neuronal damage, axonal regeneration difficulties and decreased synaptic plasticity, resulting in neurological dysfunction [3]. One critical reason is that the brain contains many types of myelin-associated inhibitory proteins (MAI) and their receptors. Studies have found that paired immunoglobulin-like receptor B (PirB) is a co-receptor of Nogo-A, myelin associated glycoprotein (MAG) and oligodendrocyte myelin glycoprotein (GP)[4–6]. It was reported that the *pirb* gene knockout mice had smaller cerebral infarction volume than the wild-type mice after focal cerebral ischemia injury [7]. These results suggest that PirB may be necessary for the aggravation of neuronal damage during cerebral ischemia–reperfusion injury. Other studies have shown that PirB plays a vital role in cognitive impairment [8]. In our previous study, we found significantly increased PirB expression in neurons in the ischemic penumbra, and PirB overexpression exacerbated neuronal apoptosis [9, 10]. These pieces of evidence suggest that PirB may be an important target for treating ischemic stroke. Inhibition of PirB function could be effective in alleviating nerve cell damage and promoting recovery of nerve function. This strategy may be more effective than a single ligand, offering new hope for the treatment of ischemic stroke.

The extracellular segment of PirB mainly contains six immunoglobulin-like domains [11]. It was found that Nogo-A and MAG could combine with their extracellular components to activate the downstream signal pathway of PirB [6]. Therefore, the recovery of neuronal function after cerebral ischemic-reperfusion injury will be facilitated if PirB function can be suppressed at the protein level. In our previous studies, we generated a transactivator of transcription-PirB extracellular peptide (TAT-PEP) that had a high affinity for MAIs and ameliorated their inhibitory effect on neurite outgrowth. Furthermore, TAT-PEP can widely distribute in the penumbra after intraperitoneal injection [9]. Subsequently, we found that TAT-PEP increased neurite outgrowth and reduced growth cone collapse after oxygen–glucose deprivation (OGD) injury [8, 9]. However, it remains to be investigated whether TAT-PEP could improve cortical neuronal survival and promote recovery of cognitive function after the transient focal cerebral ischaemia model.

In this study, the GPH-PIRB-294 lentivirus system was constructed to interfere with PirB expression and to observe the effect of inhibition of PirB expression on neuronal survival after OGD injury. We used the middle cerebral artery occlusion (MCAO) model to evaluate the effects of TAT-PEP on improving neural function and cerebral infarct volume following cerebral ischaemia–reperfusion injury using neurobehavioural and MRI imaging techniques. Through in vivo and in vitro experiments, we further analysed the effect of TAT-PEP on neuronal survival, oxidative stress, degeneration and apoptosis after cerebral ischemia–reperfusion injury and investigated the relevant mechanisms to elucidate the role and mechanism of TAT-PEP on neuronal survival during cerebral ischemia–reperfusion injury.

## Materials and Methods

### Animals

Adult male Sprague–Dawley (SD) rats (280 ± 20 g) housed under controlled conditions with a 12-h light/dark cycle, a temperature of 21 ± 2 °C and humidity of 60–70% for at least 1 week before drug treatment or surgery, purchased from the Experimental Animal Center of the Fourth Military Medical University (Xi’an, Shaanxi, China) and the Laboratory Animal Center of Xi’an Jiaotong University (Xi’an, Shaanxi, China). It was approved by the Ethics Committee for Animal Experimentation of the Fourth Military Medical University and by the Institutional Animal Care and Use Committee at Xi’an Jiaotong University. Every effort was made to minimize stress to the animals. All sample sizes for the assessment parameters were calculated to reduce the number of animals used.

### Transient Focal Cerebral Ischemia Mode

Focal cerebral ischemia was induced in the rats by middle cerebral artery occlusion (MCAO), as described previously [12, 13]. The experimental animals were randomly divided into 3 groups, with 10 animals in each group. Because the success rate of the MCAO model is about 70%, the actual number of animal samples used in each group is 8 ~ 6 (*n* = 8 ~ 6 for each group). The three groups were divided into the Sham group, the MCAO group, and the MCAO + TAT-PEP group. In the MCAO + TAT-PEP group, 1.0 mg/kg TAT-PEP was intraperitoneally injected immediately after reperfusion, and then TAT-PEP of this concentration was injected daily.

### Garcia Scores

Modified Garcia scores [14] were used to assess the neurological function of the experimental animals at 24, 48 and 72 h after MCAO. It contains 18 points, including 0–3 points for each item, as shown below. Free activity in a cage for 5 min: no activity (score 0); almost unable to move (score 1 point); able to move, but the range of movement does not reach 3 sides in the cage (2 points); the field of activity shall get at least 3 sides inside the enclosure (3 points). The symmetry of limb movement: no movement of the left limb (0 points); the left limb can move slightly (1 point); the left limb can move slowly (2 points); bilateral limb movements are symmetrical (3 points). The symmetry of forelimb (forelimb extension when lifting the tail): the left limb is unable to move (0 points); the left limb can only be slightly extended (1 point); the left side is not as active and stretched as the right side (2 points); bilateral forelimbs can be tested symmetrically (3 points). Climbing in the metal cage: none (0 points); unable to mount (1 point); the left side is slightly weak (score 2 points); can usually climb (3 points). Touch bilateral trunk reaction: none (0 points); no response on the left side (score 1 point); weak left side reaction (score 2 points); the answer is symmetrical (3 points). Whisker reaction: none (0 points); no response on the left side (score 1 point); weak left side reaction (score 2 points); the reaction is symmetrical (3 points).

### Y-maze Test

The Y-maze test was used to assess spatial learning and memory as described previously [15]. Experimental rats were placed in a Y-shaped maze (arm’s length: 16 cm, arm width: 10 cm, height of the wall: 40 cm, Yihong Technology Co., Ltd., Wuhan, China) with three arms at 120° from each other and allowed to explore the three arms for 10 min freely. Before all experiments, the rats should be used for acclimatization for 2 days, during which the rats can visit all components freely for 10 min. The number of arm entries and alterations were recorded automatically using Smart Video Tracking Software 3.0 (Panlab, Barcelona, Spain). The percentage of spontaneous alternation was calculated as the number of correct alterations (number of total new arm entries), which is associated with the capacity of spatial short-term memory.

### Novel Object Recognition Test (NOR)

The New Object Recognition Test has been widely used in the study of cognitive impairment [16]. First, to reduce stress levels, all groups of rats were placed in the experimental room and testing box for 10 min on the 2 successive days before the training phase. At 3 days post-MCAO, rats were trained to explore freely in a box (Yihong Technology Co., Ltd., Wuhan, China). The experimental method was described previously. The experiment was recorded with a video camera (SNC-VB600B5, SSGE, Shanghai, China) cation placed on the old object. Exploration time for novel objects during testing was assessed for each group.

### TTC Staining

At 3 days after MCAO, TTC staining was used to detect the effect of TAT-PEP on cerebral infarction volume. The straightforward method is as follows [17]: The brain was removed under deep anaesthesia and immersed in normal saline ice for 10 min. Put it into the cerebral sulcus mould, and the thickness of the brain slice is about 2 mm (cut in sequence along the coronal plane). Soak the brain slices in a 2% concentration of a 2,3,5-TTC solution with the same orientation, and incubate them in a constant temperature water bath (37 °C, 30 min). After the staining effect is appropriate (the white area is the infarcted area, and the red area is the normal brain tissue area), transfer to 4% PFA for fixation overnight, take photos and analyze 24 h later. Adobe Photoshop CS3 image processing software was used to calculate the volume of cerebral infarction.

### MRI Detected Cerebral Infarction Volume

Three days after MCAO, the effect of TAT-PEP on cerebral infarct volume was assessed using small animal MRI. The simple method follows: After anaesthesia, the rats were placed in a prone position with their heads in the centre of the coil. The rapid acquisition relaxation enhancement sequence (along the coronal section) was used to scan with the visual intersection as the origin. The parameters are an echo time (TE) of 60 ms, a repetition time (TR) of 3000 ms, a thickness of 0.5 mm and no spacing. The field of view is 2.56 cm × 2.56 cm. Acquire T2-weighted (T2WI) images, and then use image software for image analysis to measure the volume of ischemic injury area (i.e. abnormal high signal area), which is equal to the area of abnormally high signal area × the sum of slice thickness (plus spacing), used to evaluate the cerebral infarction volume.

### Nissl Staining

Nissl staining (Beyotime Institute of Biotechnology, China) was performed to detect Nissl bodies in the cytoplasm of surviving neurons. At 3 days after MCAO, Nissl staining was used to detect the effect of TAT-PEP on the survival of neurons in the penumbra. The methods are described as follows: Dry the slices at room temperature for 1 h, draw circles and gently rinse them with 0.01 M PBS 3 times (5 min/time). Add Nissl dyestuff (to filter in advance) and incubate at 37 °C for 20 min. Then, gently wash off with pure water. The sections were dehydrated and transparent before using the neutral resin sealed. The microscope was used to observe and record in the open field. The integrated optical density/area of the staining in each group was acquired by 2 blinded investigators using ImagePro Plus 5.1 software (Media Cybernetics, Inc., Bethesda, MD).

### FJC Staining

At 3 days after MCAO, FJC staining was used to detect the effect of TAT-PEP on the number of degenerated neurons in the penumbra. The specific methods are as follows: Freeze the slices and dry them at room temperature for 1 h, draw circles and gently rinse them with 0.01 M PBS 3 times (5 min/time). Immerse the slices in a mixture of 1% sodium hydroxide and 80% anhydrous ethanol for 5 min. Then, immerse it in 70% absolute ethanol, hydrate it for 2 min and gently rinse it with deionized water 3 times (5 min/time). Incubate with 0.06% potassium permanganate for 10 min (shaking table at room temperature) and rinse with distilled water for 5 min (shaking table). Prepare 0.0001% FJC dye solution and add acetic acid in the proportion of 1:1000. After dropping FJC dye solution, incubate it at 37 °C at constant temperature and away from light for 30 min, and gently rinse it with deionized water 3 times (1 min/time). Dry in the dark at room temperature, dehydrated with anhydrous ethanol for 2 min, make xylene transparent 3 times (2 min/time) and apply neutral gum film. The people who did not know the experimental group were observed by Olympus BX51 fluorescence microscope (excitation light wavelength was 450–490 nm), collected images and recorded the number of FJC positive cells (FJC positive cells were green).

### Immunofluorescence Histochemical Staining

On the 3rd day after MCAO, NeuN immunofluorescence histochemistry was used to detect the effect of TAT-PEP on the number of living neurons in the penumbra. The specific method is the same as that in Part I. First antibody (NeuN, 1:5000) was added, 4 °C overnight, 0.01 M PBS solution was used for rinsing (5 min each time × 3 times), inject the secondary antibody (goat anti-mouse FITC, 1:5000), incubated at room temperature in the dark for 2 h, rinse in the night for 3 times, observe and take photos under a fluorescence microscope after 50% glycerine film is sealed.

### TUNEL Staining

At the third day after MCAO, TUNEL staining was used to detect the effect of TAT-PEP on the number of apoptotic neurons in the penumbra. The specific methods are as follows: The fixed brain tissue was immersed in 30% alcohol, embedded in paraffin and sliced, with a thickness of 3 μm. Paraffin sections were dewaxed with xylene for 5 min (2 min/time). After waxing, use a circle drawing brush to draw circles, drip protein kinase K cell permeating solution, incubate at 37 ℃ for 30 min and then rinse gently with 0.01 M PBS 3 times (5 min/time). Join 50 μL DNase1 solution at room temperature for 10 min. After the slides are cleaned and dried, add the TUNEL mixture, 37 ℃, 1 h PBS cleaning again. Joining 30 μL POD stops the reaction, and PBS has cleaned again. Drop the DAB colour-developing solution at room temperature for 10 min, and then conduct PBS cleaning again. Drop haematoxylin for 30 s and gently wash it under pure water. Gradient dehydration, xylene transparent for 5 min (twice), neutral resin film, open field microscope observation and statistics of the number of TUNEL-positive cells (TUNEL-positive cells are brown black).

Experimental animals were anaesthetised intraperitoneally with 0.3 mL/100 g chloral hydrate (300 mg/kg), perfused and fixed with a solution containing 4% PFA and 0.05% glutaraldehyde, positioned on a stereotaxic device and fixed for 2 h. Coronal sections were made with a vibrating microtome with a thickness of 50 μm and immersed in PBS solution containing 30% sucrose for 2 h. Dehydrate with gradient alcohol and put the transparent continuous sections on copper mesh with supporting membrane, and observe the ultrastructure of neurons under the electron microscope.

### Primary Culture of Cortical Neurons

Primary cortical neurons were cultured as described previously [17]. SD rats who were pregnant 16.5–18.5 days (E 16.5–18.5 days) were dislocated and killed. A routine aseptic operation was carried out. Foetal rats were taken out and separated. In the D-Hank’s (HyClone,SH30022.01) solution (placed on ice), the foetal rat was decapitated with toothless ophthalmic tweezers, and the brain tissue was taken out. Under the microscope, the cerebral cortex was separated. The tissue was chelated through D-Hank’s solution. Then, trypsin (0.125%) was digested for 15 min (in a regular incubator at 37 °C). Then, the tissue was carefully sucked out with an elbow dropper, added into a centrifuge tube (15 mL) containing DMEM (Solarbio, H1045) solution of foetal bovine serum, and the digestion was stopped at room temperature for 5 min. Place the centrifuge tube in the centrifuge at 80 rpm for 5 min, discard the supernatant carefully, add 2 mL of DMEM solution containing foetal bovine serum, blow and repeatedly beat to prepare tissue and cell suspension, and use the cell filter (100 μm) filtering. After another centrifugation at 80 rpm for 5 min, it was added to the neuron culture medium (Neurobasal A, 2% B27, 1% glutamate, and 1% penicillin mixture). According to the experimental requirements, different-density cells were inoculated into 96-well plates, 24-well plates or 6-well plates coated with poly-l-lysine (50 mg/mL) (Sigma, USA). These cells were grown in Neurobasal medium (Gibco, Invitrogen Corp, USA) supplemented with 2% B27, 1% glutamine and 1% penicillin/streptomycin (Sigma, USA) at 37 °C under a humidified incubator in air containing 5% CO_2_. The purity of neurons was determined by immunocytochemistry for βIII-tubulin at 5 days after plating, which indicated that 95% of the cells in cultures were positive for βIII-tubulin (1:250; Millipore, Temecula, CA, USA) (data not shown).

### Oxygen Glucose Deprivation Model Of Neurons In Vitro

Sugar-free and serum-free culture medium was replaced and placed into hypoxia constant temperature incubator (37 °C, 5% CO_2_, 95% N2) for 1 h. Culture media in the culture dish or culture bottle/flask was replaced with standard neuron culture medium (Neurobasal A, 2% B27, 1% glutamate, and 1% penicillin mixture) and transferred into traditional incubator (5% CO_2_, 21% O_2_, 37 °C).And then, they were immediately given TAT-PEP or TAT mPEP. The experimental groups are as follows:
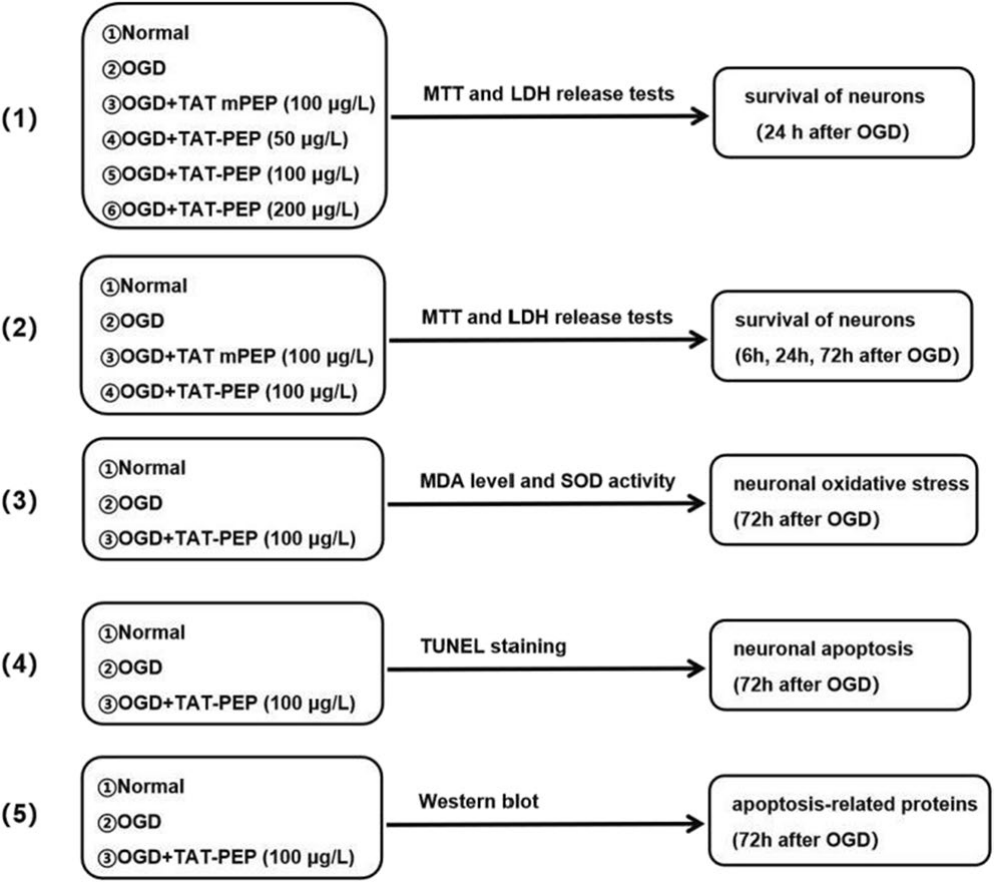


After primary cerebral cortical neurons grew well in 24 h, cells were divided into four groups as follows: Normal, OGD, OGD + Con RNAi and OGD + PirB RNAi groups. Cells in the OGD + Con RNAi and OGD + PirB RNAi groups were given the neurobasal medium with lentiviral shRNA vectors for 12 h, and cultured in normal neurobasal medium. Thus, these cells were transiently transfected with Con RNAi construct or PirB RNAi construct, which also expressed GFP. PirB, GFP and β-actin were detected in extracts by immunofluorescence and western blot analysis at 3 days after infection.

### Overexpression PirB in Cortical Neurons

Primary cortical neurons were separated. After 7 days of culture, cells were transfected with CRISPR-PirB activation or CRISPR-Ctrl (Santa Cruz Biotechnology). After 48 h, transfected cells were exposed to OGD.

### qRT-PCR

Cells were treated with TRIzol reagent (Life Technologies, Carlsbad, CA, USA, no. 15596018) according to the manufacturer’s protocols. A First-Strand cDNA Synthesis Kit (Promega) was used to reverse transcripts. The cDNA was diluted to 10 times with ddH_2_O. Then, cDNA from various groups was quantified by using Fast SYBR Green Master Mix (Thermo Fisher) and a Bio-Rad qRT-PCR System (Roche, Basel, Switzerland). Primers for PirB were: 5′-TACAAGGAAGTACCACGCCC-3′ (forward) and 5′-GGTTCAGCCTTGATGGTTGG-3′ (reverse). The relative gene expression level was normalized to that of β-actin [18, 19].

### Western Blot

According to the time node of the experiment, the whole cell protein was extracted. The method follows: After washing PBS three times, add precooled Lysis cracking solution (100 μL/well), carefully scrape the cells and thoroughly mix the lysate with the cells. Transfer the suspension into the precooled EP tube, ice bath for 30 min and centrifugation at 4 °C for 15 min, 12,000 rpm. The total protein concentration of the tissues or cells was analysed with a BCA kit (Sigma, CA, USA). Rabbit antibody against cleaved (active) caspase-3 (1:1,000; Cell Signaling Technology, Beverly, MA, USA), mouse monoclonal antibodies against Bcl-2 or Bax (1:1,000, Santa Cruz, CA, USA) and rabbit antibody against PriB (1:1,000, Abcam, USA), and β-action (1:2,000, Anbo, USA). Subsequently, the blots were probed with horseradish peroxidase (HRP)-conjugated goat secondary antibody against rabbit or mouse IgG (1:1,000, Abcam, USA). Detection and quantitation were performed with a Typhoon 9400 Variable Mode Imager (GE Healthcare) and Lumi-Light Western Blotting Substrate (Roche Diagnostics) for HRP-labelled blots.

### TUNEL Staining

TUNEL staining was performed in vitro using an In Situ Cell Death Detection Kit (Roche Diagnostics, Mannheim, Germany). At 72 h after OGD injury, 4% PFA was added to fix at room temperature for 1 h. PBS (0.01 M) was gently rinsed 3 times (5 min/time), and then 0.3% hydrogen peroxide was used for 10 min. Add TUNEL reaction mixture for 1 h at 37 °C and stain with DAPI for 5 min at room temperature. Images were obtained with a microscope (BX60, Olympus). The integrated optical density/area of the positive TUNEL staining in each group was acquired as described above.

### Statistical Analysis

All data are presented as the mean ± SD across the groups and were statistically analysed using GraphPad Prism 7.0 software (GraphPad Company, San Diego, CA, USA). Continuous data were tested for normal distribution and analysed by one-way ANOVA (followed by Tukey’s multiple comparisons tests) or Kruskal–Wallis test (followed by Dunn’s multiple comparisons tests). *P* value of less than 0.05 was considered statistically significant.

## Results

### TAT-PEP Improved Neurobehavioral Function and Cognitive Function Against MCAO

**Fig. 1:**
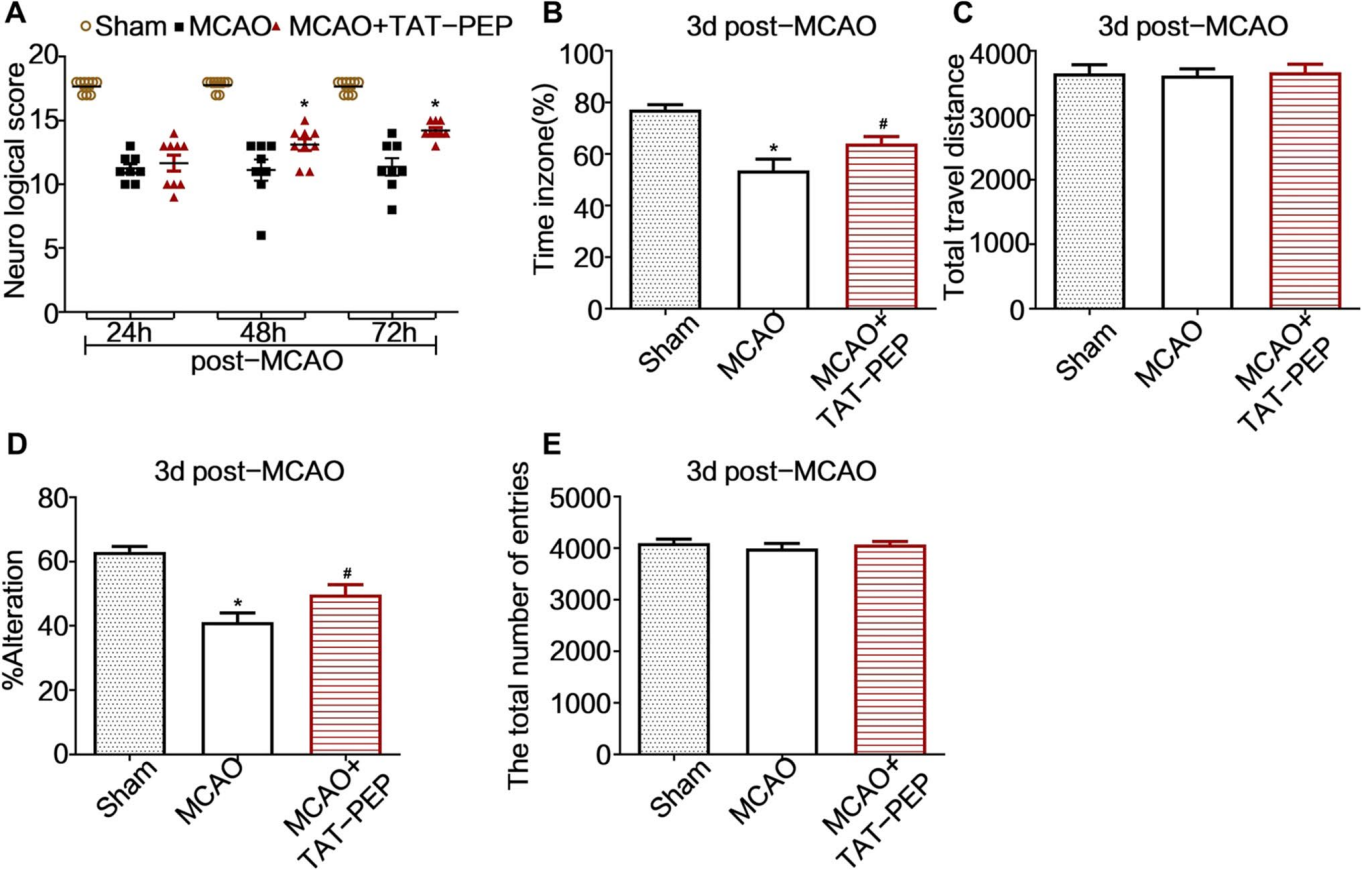
TAT-PEP enhanced neurobehavioral function and cognitive function recovery. **A** Garcia scores tested the effect of TAT-PEP on neurobehavioral function recovery of experimental animals in each group at 24 h, 48 h and 72 h after MCAO (*n* = 8, each group; *P < 0.05 vs. sham group; #*P* < 0.05 vs. MCAO group). **B**, **C** The recognition index and total travel distance in the novel object recognition test were measured (*n* = 6). **D**, **E** Percent of spontaneous alteration and total arm entries in the Y-maze test were measured (*n* = 6)

We used Garcia scores to analyze neurobehavioral function recovery. As shown in Fig. 1A, the scores of the TAT-PEP treatment group were higher than that of the Sham group at 48 h (*P* < 0.05) and 72 h (*P* < 0.05) after reperfusion. We used the Y-maze test to analyze cognitive function recovery. At 3 days after reperfusion, the per cent of spontaneous alteration and total arm entries in the Y-maze test were measured. Rats of the MCAO group showed significantly lower spontaneous alternation rates than rats of the Sham group in the Y maze test.

In contrast, in the Y maze test, rats of the MCAO + TAT-PEP group showed substantially higher spontaneous alternation rates than rats of the MCAO group (Fig. 1B). Also, the recognition index and total travel distance in the novel object recognition test were measured. Rats of the MCAO group spent less time exploring a novel object during the test phase than the Sham group rats. In contrast, the MCAO + TAT-PEP group spent more time exploring a novel thing during the test phase than the rats of the MCAO group (Fig. 1D). These results indicated that TAT-PEP could promote neurological and cognitive functional recovery.

### TAT-PEP Reduced Brain Infarct Volume Against MCAO

**Fig. 2:**
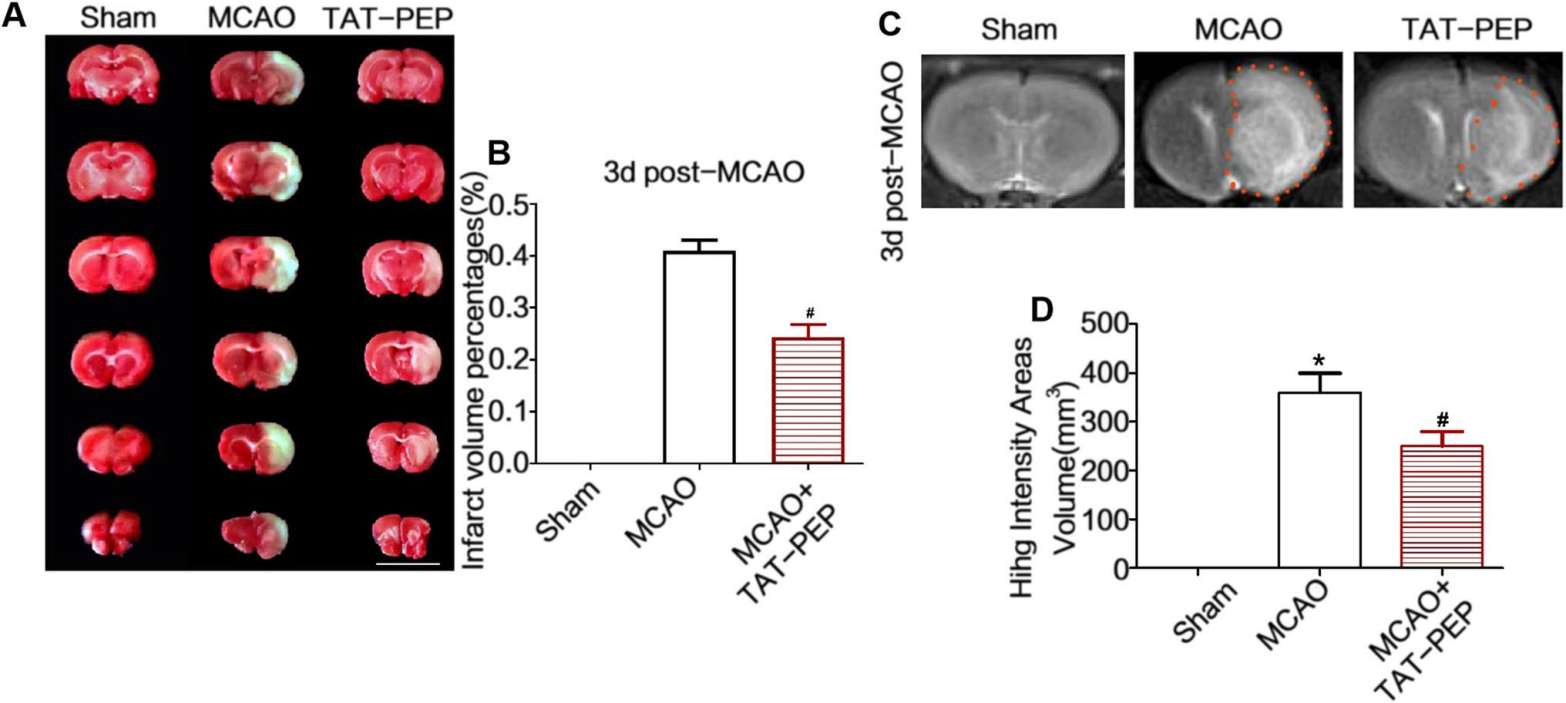
TAT-PEP reduced the volume of cerebral infarction. **A** TTC staining detected the volume of cerebral infarction (Bar = 1 cm; *n* = 6, each group). The picture of TTC staining of representative brain slices of experimental animals in each group at 3d post-MCAO. **B** It was the statistical result of cerebral infarction volume percentage in each group (*P < 0.05 vs. sham group; #*P* < 0.05 vs. MCAO group). **C** Magnetic resonance imaging detected the effect of TAT-PEP on the cerebral infarction volume of experimental animals in each group at 3d post-MCAO (*n* = 6, each group). It was the T2WI signal imaging result. **D** The statistical analysis of T2WI signal imaging on cerebral infarction volume (*P < 0.05 vs. Sham group; #*P* < 0.05 vs. MCAO group)

Then, we tested the brain infarct volume in every group. Figure 2A and B show that the MCAO + TAT-PEP group displayed a significantly smaller brain infarct volume than the MCAO group by TTC staining at 3 days post-MCAO (*P* < 0.05). MRI measured infarction volumes. T2WI analyses showed that the high-intensity books were more prominent in the MCAO group than in the Sham group at 3 days post-MCAO. In contrast, T2WI studies showed that the high-intensity volumes were smaller in the MCAO + TAT-PEP group than in the MCAO group at 3 days post-MCAO (*P* < 0.05).

### TAT-PEP Attenuated Neuronal Degeneration, Apoptosis, Mitochondria Damage and Oxidative Stress Against MCAO

**Fig. 3:**
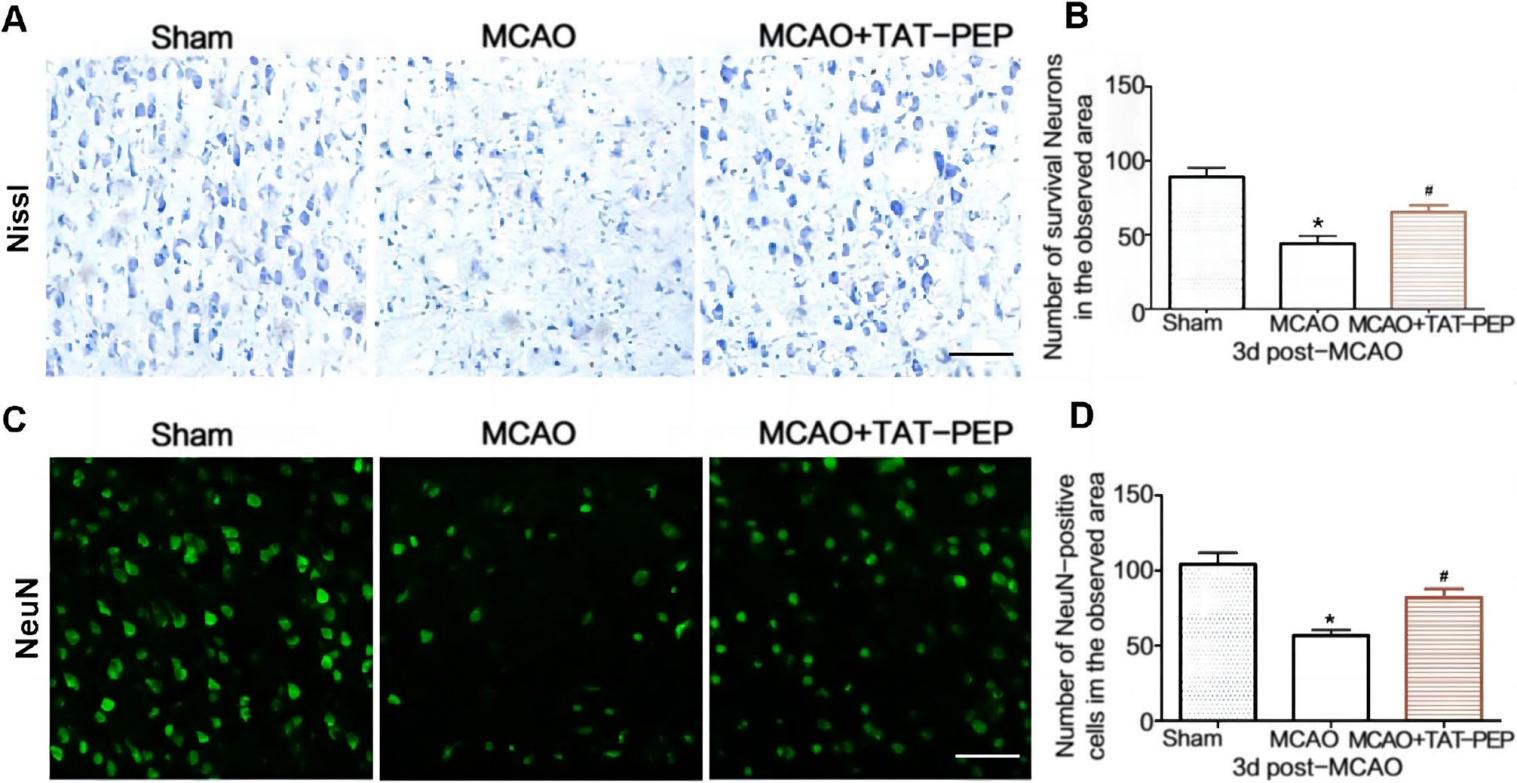
The effect of TAT-PEP on the survival of neurons in the penumbra (*n* = 6, each group). **A** Nissl staining detected the number of living neurons in the penumbra of each group at 3d post-MCAO (Bar = 50 µm). **B** It was the statistical graph of the number of survival neurons in the penumbra of each group at 3d post-MCAO (*P < 0.05 vs. sham group; #*P* < 0.05 vs. MCAO group). **C** Immunofluorescence histochemical staining detected the number of NeuN-positive neurons (green) in the penumbra of each group at 3d post-MCAO (Bar = 50 µm). **D** It was the statistical graph of the number of NeuN-positive neurons in the penumbra of each group at 3d post-MCAO (*P < 0.05 vs. Sham group; #*P* < 0.05 vs. MCAO group)

**Fig. 4:**
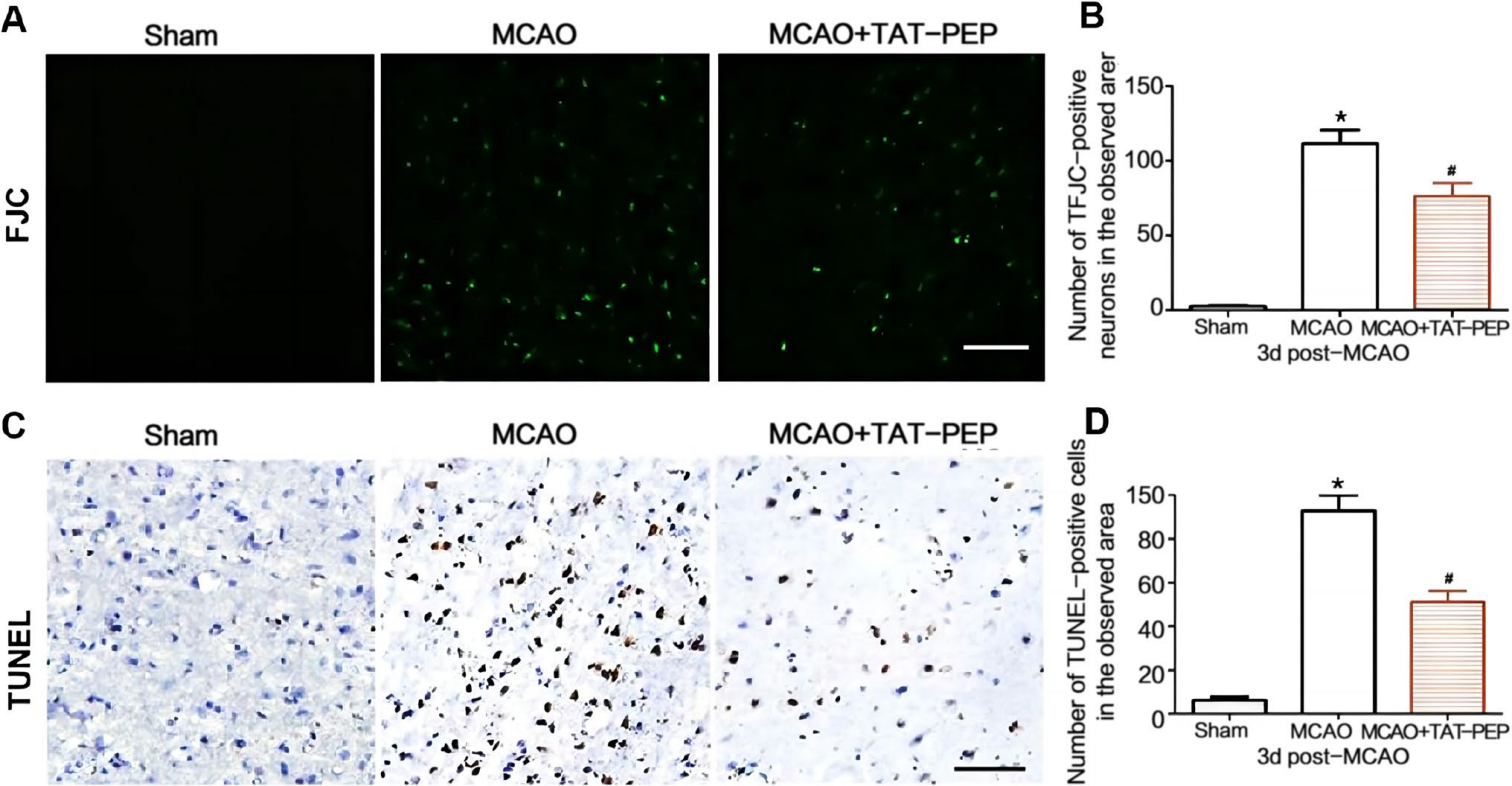
Effects of TAT-PEP on neuronal degeneration and apoptosis in the penumbra (*n* = 6, each group). **A** JFC staining detected the number of degenerated neurons in the penumbra of each group at 3d post-MCAO. The JFC-labelled degenerated neurons were green (Bar = 50 µ m). **B** It was the statistical graph of the number of degenerated neurons in the penumbra of each group at 3d post-MCAO (*P < 0.05 vs. Sham group; #*P* < 0.05 vs. MCAO group). **C** TUNEL staining detected the number of apoptotic neurons in the penumbra of each group at 3d post-MCAO. TUNEL-positive neurons were brown and black (Bar = 50 µm). **D** It was the statistical graph of the number of TUNEL positive neurons in the penumbra of each group at 3d post-MCAO (*P < 0.05 vs. Sham group; #*P* < 0.05 vs. MCAO group)

**Fig. 5:**
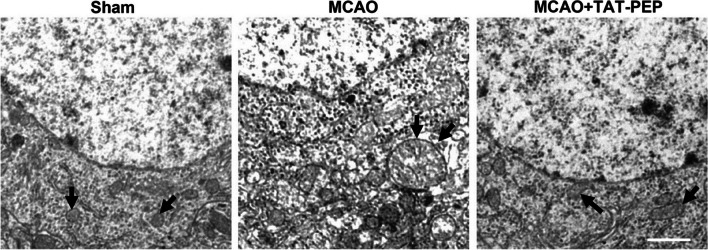
Effects of TAT-PEP on the ultrastructure of neurons in the penumbra (*n* = 6, each group). Electron microscope observed the ultrastructure of neurons in the penumbra of each group 3 days after MCAO. Arrows indicated swollen mitochondria in the cytoplasm. Mitochondria expand into spheres. Bar = 2 µm

To assess neuroprotection of TAT-PEP, the Nissl staining and NeuN staining were performed in the ischemic penumbra at 3 days after reperfusion. Compared with the MCAO group, the density of normal neurons in the ischemic penumbra in the MCAO + TAT-PEP group increased significantly (*P* < 0.05) (Fig. 3A, B). The number of NeuN-positive neurons was more in the MCAO + TAT-PEP group than that in the MCAO group (*P* < 0.05) (Fig. 3C, D). Then, the FJC and TUNEL staining on ischemic brain sections was performed at 3 days after reperfusion. The number of FJC-positive neurons was fewer in the TAT-PEP group than in the MCAO group (*P* < 0.05). Similarly, the number of TUNEL-positive cells was more irregular in the TAT-PEP group than that in the MCAO group (*P* < 0.05) (Fig. 4A–D). At 3 days after reperfusion, the ultrastructure of cortical neurons in the MCAO group showed nuclear membrane folds, collapse and blurred boundaries. Additionally, noticeable swelling of mitochondria in the cytoplasm was noted, which was spherical. Some mitochondrial cristae were fractured. The damage to the neuronal ultrastructure after TAT-PEP treatment was significantly alleviated, especially in the mitochondria (Fig. 5).

**Fig. 6:**
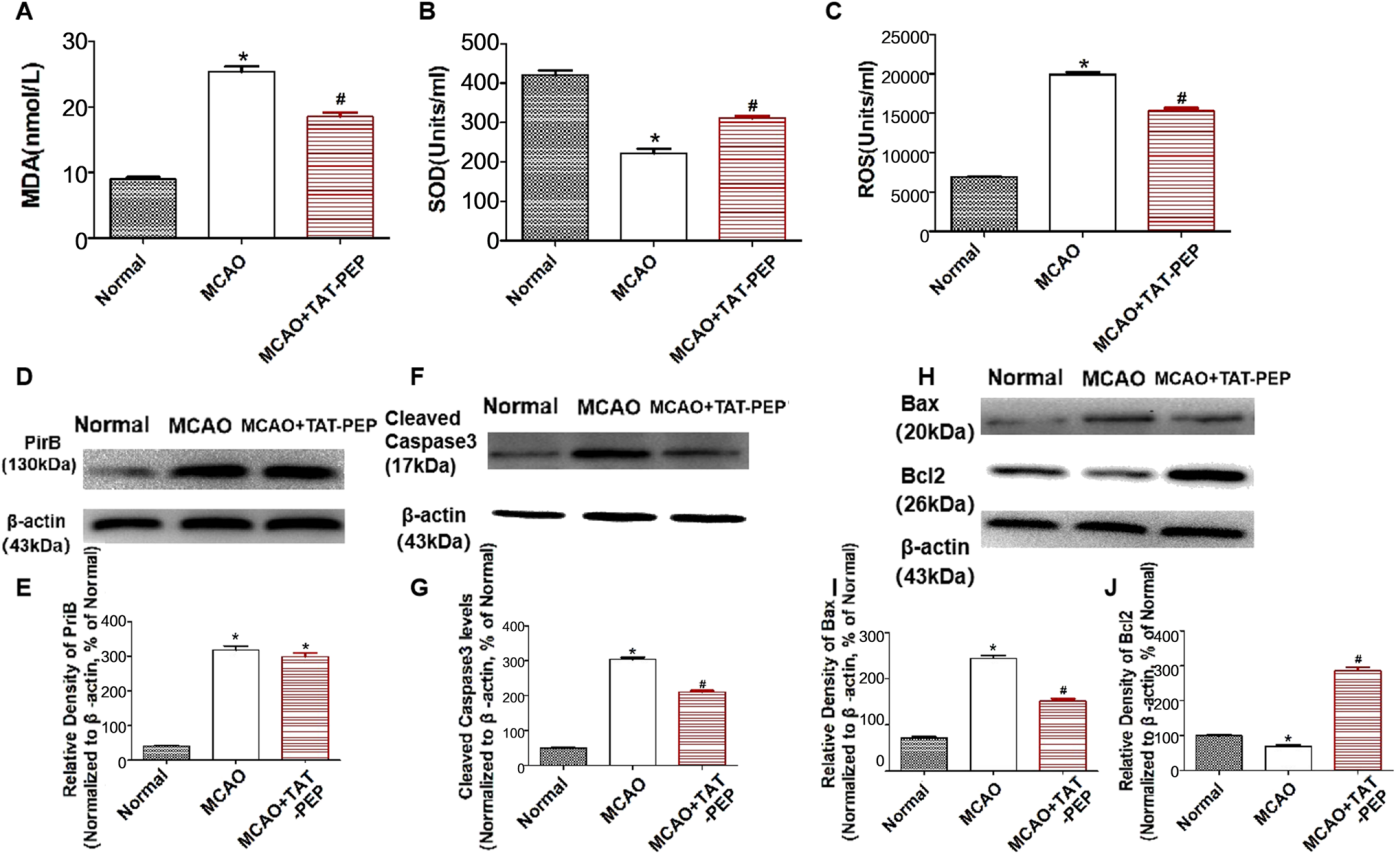
**A**, **B**, **C** The effect of TAT-PEP on MDA level, SOD activity and ROS accumulation in each group at 3d post-MCAO (*n* = 6, each group). **D**, **F**, **H** The protein band of PriB, Cleaved Caspase3, Bax and Bcl2 expression in every group at 3d post-MCAO was detected by western blot. **E**, **G**, **I**, **J** The statistical diagrams of PriB, Cleaved Caspase3, Bax and Bcl2 expression in every group at 3d post-MCAO (*P < 0.05 vs. Normal group; #*P* < 0.05 vs. MCAO group)

Malondialdehyde (MDA), superoxide dismutase (SOD) and reactive oxygen species (ROS) are regarded as indicators for detecting oxidative stress. The level of MDA, SOD and ROS in ischemic penumbra was performed at 3 days after reperfusion. The MDA and ROS content in the MCAO group increased compared with that in the normal group (*P* < 0.05). TAT-PEP treatment attenuated the elevation of MDA and ROS content compared with that in the MCAO group (*P* < 0.05) (Fig. 6A, C). Compared with the normal group, SOD activity was reduced in the MCAO group (*P* < 0.05). TAT-PEP treatment increased the SOD activity compared with that in the MCAO group (*P* < 0.05) (Fig. 6B). The expression of PriB was assessed by western blot at 3 d after reperfusion. The level of PriB was significantly increased in the MCAO group and MCAO + PEP group compared with the normal group (*P* < 0.05) (Fig. 6D, E). The levels of PriB did not show any significant difference between the MCAO group and the MCAO + PEP group. The expression of cleaved (active) Caspase3, Bcl-2 and Bax was assessed by western blot at 3 days after reperfusion. Caspase3 activity was significantly inhibited in the MCAO + PEP group compared with the MCAO group (*P* < 0.05). Treatment with TAT-PEP increased the level of Bcl-2 protein, whereas it decreased Bax expression compared with the MCAO group (*P* < 0.05) (Fig. 6F–J).

### Inhibiting PirB Overexpression was Beneficial to Rescue Neurons from Apoptosis Exposed to OGD Injury

**Fig. 7:**
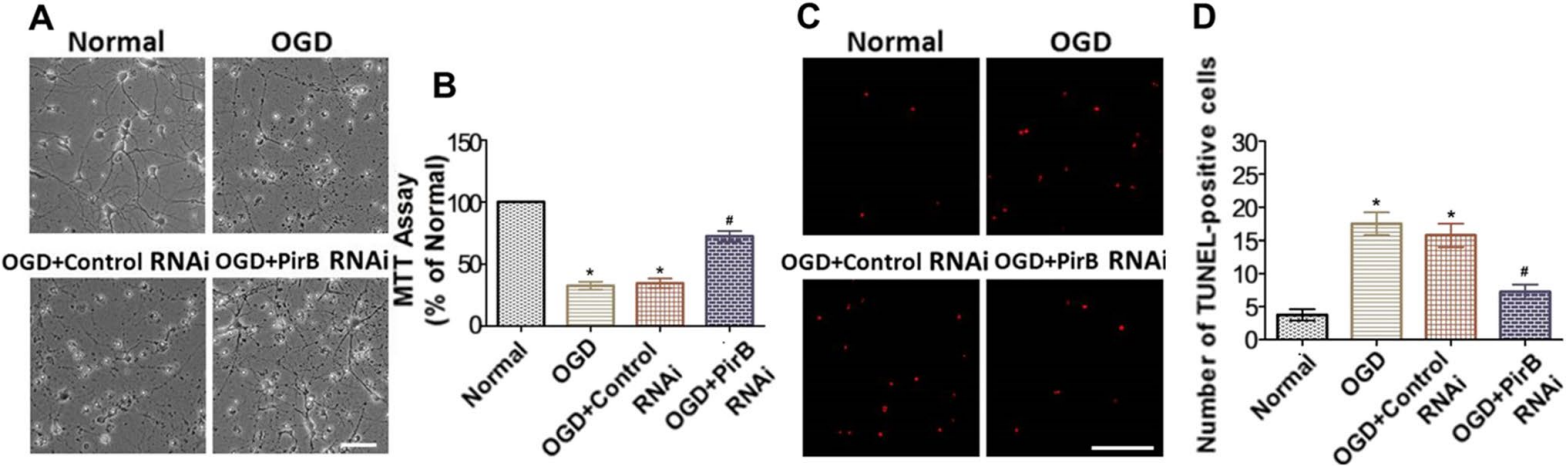
Effect of *pirb* RNAi on neuronal survival and apoptosis exposed to OGD injury (*n* = 6, each group). **A** It was to observe the survival status of neurons in 293 T cells transfected with GPH-PIRB-294 lentivirus for 72 h under a bright field microscope, Bar = 50 μm. **B** It was the statistical diagram of MTT analysis of neuronal activity 72 h after GPH-PIRB-294 lentivirus transfected neurons to OGD (*P < 0.05 vs. Normal group; #*P* < 0.05 vs. OGD group). **C** GPH-PIRB-294 lentivirus was transfected into the OGD of neurons, and then GPH-PIRB-294 was transfected 72 h after OGD; TUNEL staining was used to observe the apoptosis of neurons. Red cells are TUNEL-positive neurons, Bar = 100 μ m. **D** It was the statistical graph of TUNEL-positive neurons in each group (*P < 0.05 vs. normal group; #*P* < 0.05 vs. OGD group)

The survival of cultured neurons was examined at 72 h after PirB RNAi vector or control RNAi transfection. The neuronal structure in the OGD group and OGD + control RNAi group was damaged. However, transfection with the PirB RNAi vector prevented neuronal damage. The cell viability in the OGD and control RNAi groups was significantly lower than that of the normal group (*P* < 0.05) at 72 h after exposure to OGD. However, the cell viability of the PirB RNAi group was higher than that of the OGD and control RNAi groups (*P* < 0.05) (Fig. 7A, B). The number of TUNEL-positive cells in the OGD and Control RNAi groups was significantly higher than that of the normal group (*P* < 0.05) at 72 h after exposure to OGD. In addition, the number of TUNEL-positive cells in the PirB RNAi group was less than that of the OGD group (*P* < 0.05) (Fig. 7C, D). These results indicated that inhibiting PirB overexpression was beneficial in rescuing the neurons from apoptosis.

### Over-expression of PirB Decreased the Survival of Neurons and Accelerated Neuronal Apoptosis

**Fig. 8:**
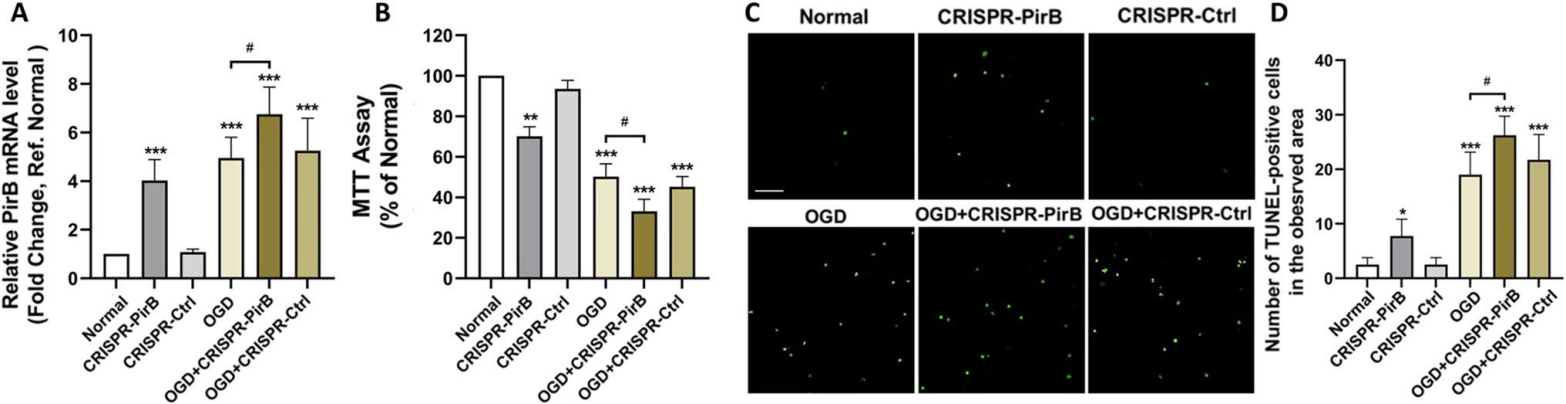
Effect of over-expression of PirB on neuronal survival and apoptosis (*n* = 4, each group). **A** It was the results of qPCR to observe the relative mRNA level of PirB in each group. **B** It was the statistical diagram of MTT analysis of neuronal activity. **C** TUNEL staining was used to observe the apoptosis of neurons. Green cells were TUNEL-positive neurons, bar = 100 μ m. **D** It was the statistical graph of TUNEL-positive neurons in each group (*P < 0.05 vs. normal group; **P < 0.05 vs. normal group; ***P < 0.05 vs. normal group; #*P* < 0.05 vs. OGD group)

We studied the impact of PirB over-expression on neuronal survival and apoptosis. The results from the qRT-PCR showed that the expression level of PirB in CRISPR-PirB activation group, OGD group, OGD + CRISPR-PirB activation group and OGD + CRISPR-Ctrl group were more than that in normal group (*P* < 0.001). The results from the qRT-PCR also showed that the expression level of PirB in OGD + CRISPR-PirB activation group was more than that in OGD group (*P* < 0.05) (Fig. 8A). We observed the over-expression of PirB on neuronal survival. The cell viability decreased significantly in CRISPR-PirB activation group (*P* < 0.01), OGD group (*P* < 0.001), OGD + CRISPR-PirB activation group (*P* < 0.001) and OGD + CRISPR-Ctrl group (*P* < 0.001) than that in the normal group. The result also showed that the cell viability in OGD + CRISPR-PirB activation group was lower than that in OGD group (*P* < 0.05) (Fig. 8B). In addition, the number of TUNEL-positive cells in CRISPR-PirB activation group (*P* < 0.05), OGD group (*P* < 0.001), OGD + CRISPR-PirB activation group (*P* < 0.001) and OGD + CRISPR-Ctrl group (*P* < 0.001) was more than that in the normal group. The result also showed that the number of TUNEL-positive cells in OGD + CRISPR-PirB activation group was more than that in OGD group (*P* < 0.05) (Fig. 8C, D).

### TAT-PEP Enhanced the Survival of Neurons Exposed to OGD Injury

**Fig. 9:**
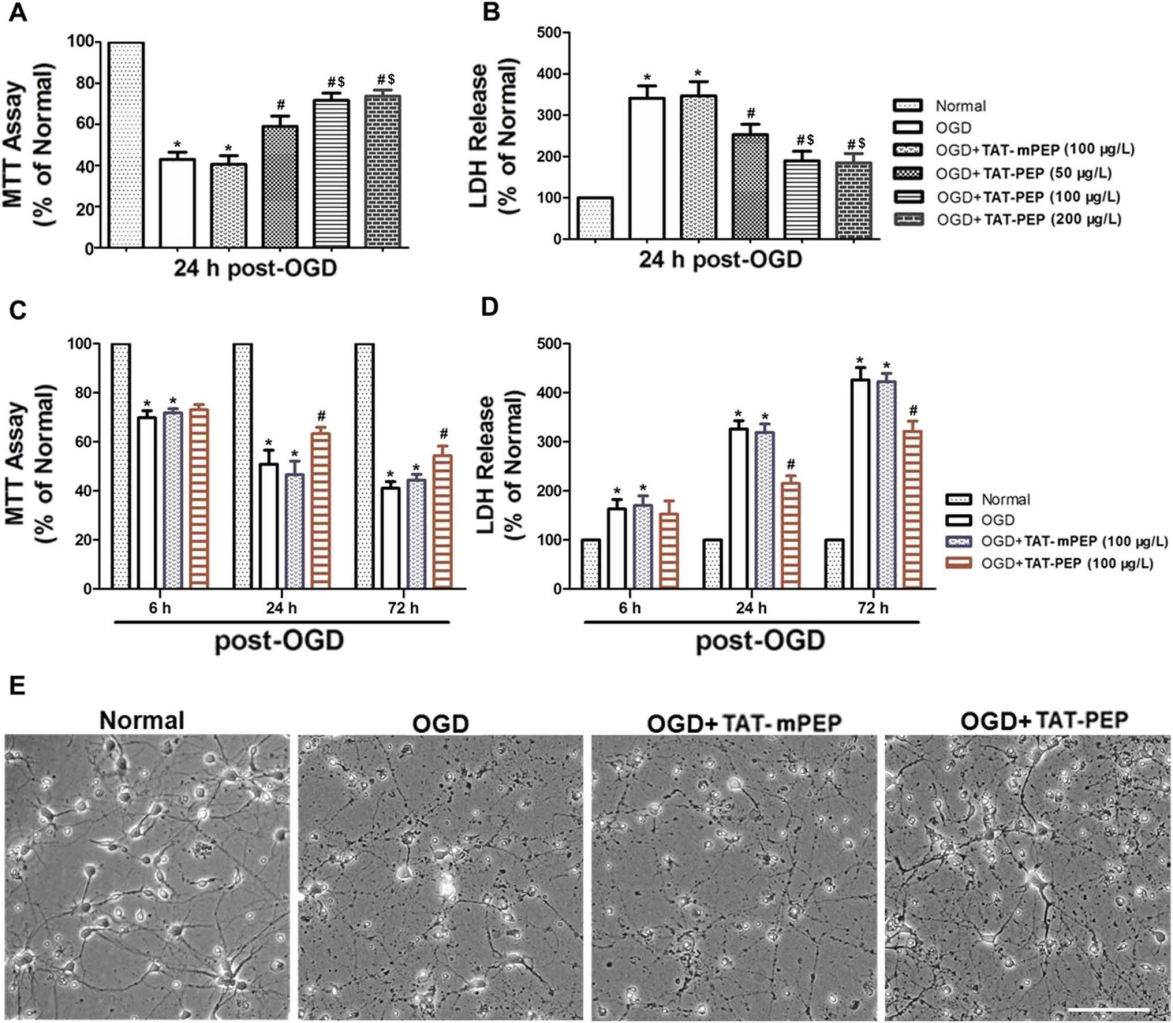
The effect of TAT-PEP on neuronal viability and survival exposed to OGD injury (*n* = 6, each group). **A** It was an MTT test to detect the impact of TAT-PEP of different concentrations on neuronal activity (*P < 0.05 vs. normal group; #*P* < 0.05 vs. OGD group; $*P* < 0.05 vs. OGD + TAT-PEP (100 μg/L) group). **B** It was LDH release test to detect the effect of TAT-PEP of different concentrations on neuronal survival (*P < 0.05 vs. normal group; #*P* < 0.05 vs. OGD group; $*P* < 0.05 vs. OGD + TAT-PEP (100 μg/L) group). **C** MTT assay detected the effect of TAT-PEP (100 μg/L) on the neuronal activity at different time points after OGD injury (*P < 0.05 vs. normal group; #*P* < 0.05 vs. OGD group). **D** LDH release test detected the effect of TAT-PEP (100 μg/L) on neuronal survival at different time points after OGD injury (*P < 0.05 vs. normal group; #*P* < 0.05 vs. OGD group). **E** Observed the effect of TAT-PEP (100 μg/L) on neuronal survival status by Brightfield microscope at 72 h after OGD injury. Bar = 100 μm

Here, we observed the function of different doses of TAT-PEP on neuronal survival at 24 h after being exposed to OGD (Fig. 9A, B). The cell viability decreased significantly in the OGD group compared to the normal group (*P* < 0.05), and LDH release (*P* < 0.05) increased substantially in the OGD group compared to the normal group (*P* < 0.05) at 24 h after exposure to OGD. In addition, the cell viability in TAT-PEP (50 μg/L, 100 μg/L, 200 μg/L) treatment group was significantly higher than it was in the OGD group or TAT-mPEP (misordered sequences of the extracellular cDNA of PirB) group at 24 h after exposure to OGD (*P* < 0.05). The LDH release in the TAT-PEP treatment group was significantly lower than that in the OGD group at 24 h after being exposed to OGD (*P* < 0.05). The results further showed that compared with the 50-μg/L TAT-PEP treatment group, the cell viability was higher, and LDH release was lower in the 100 μg/L and 200 μg/L TAT-PEP treatment groups (*P* < 0.05). However, the effect of TAT-PEP between 100 μg/L and 200 μg/L had no difference in cell survival (*P* > 0.05).

In addition, to confirm the protective effect of TAT-PEP, we also observed the function of TAT-PEP on neurons at different time points after being exposed to OGD (Fig. 9C, D). The cell viability decreased significantly in the OGD group or TAT-mPEP group compared to the normal group (*P* < 0.05), and LDH release (*P* < 0.05) increased considerably in the OGD group or TAT-mPEP group compared to the normal group at 6 h (*P* < 0.05), 24 h (*P* < 0.05) and 72 h (*P* < 0.05) after exposure to OGD. However, TAT-PEP treatment increased cell viability and decreased LDH release compared to the OGD group or TAT-mPEP group at 24 h (*P* < 0.05) and 72 h (*P* < 0.05) after exposure to OGD.

At 24 h after OGD injury, the growth state of neurons was observed under the light microscope. It was found that the integrity of neurons in the OGD group was poor. The cell bodies were atrophied or broken. The neurites were broken or disappeared. However, TAT-PEP treatment could significantly improve the growth of neurons (Fig. 9E). The above results indicate that TAT-PEP can substantially enhance the activity of neurons, reduce neuronal damage and promote neuronal survival.

### TAT-PEP Decreased MDA Levels, Increased SOD Activity, and Alleviated ROS Accumulation of Neurons Exposed to OGD Injury

**Fig. 10:**
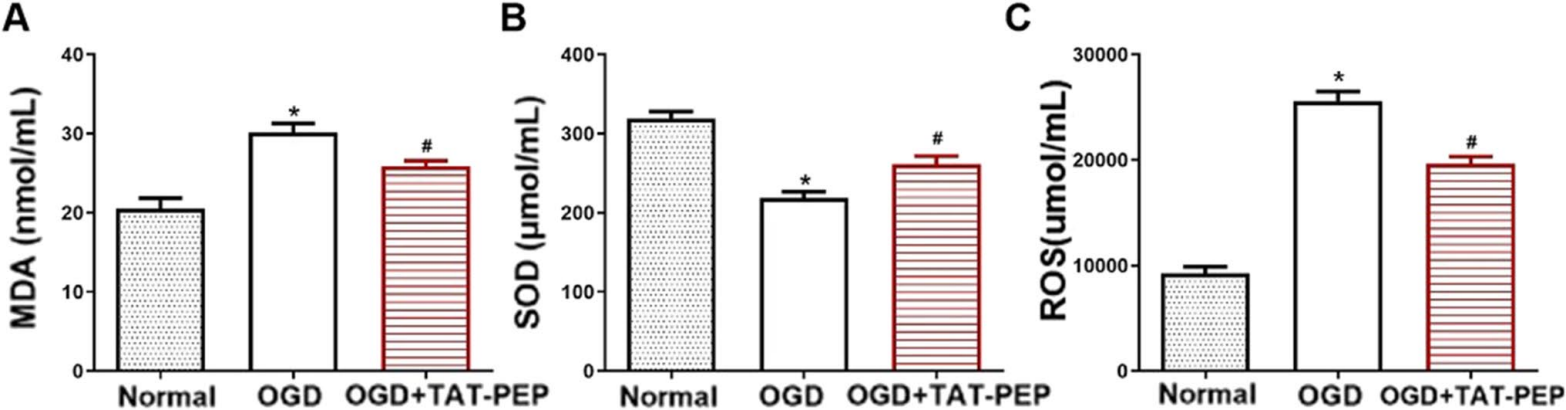
The effect of TAT-PEP on MDA level, SOD activity and ROS accumulation of neurons exposed to OGD injury (*n* = 6, each group). **A** The MDA level evaluated at 72 h after exposure to OGD injury (*P < 0.05 vs. normal group; #*P* < 0.05 vs. OGD group). **B** The SOD activity evaluated at 72 h after exposure to OGD injury (*P < 0.05 vs. normal group; #*P* < 0.05 vs. OGD group). **C** ROS production was analysed by quantitative analysis by flow cytometry at 72 h after exposure to OGD injury (*P < 0.05 vs. normal group; #*P* < 0.05 vs. OGD group)

MDA, SOD and ROS are regarded as indicators for detecting oxidative stress. The MDA content in the OGD group increased compared with that in the normal group at 72 h exposed to OGD injury (*P* < 0.05). TAT-PEP treatment attenuated the elevation of MDA content compared with that in the OGD group at 72 h exposed to OGD injury (*P* < 0.05) (Fig. 10A). Compared with the normal group, SOD activity was reduced in the OGD group at 72 h exposed to OGD injury (*P* < 0.05). TAT-PEP treatment increased the SOD activity compared with that in the OGD group at 72 h exposed to OGD injury (*P* < 0.05) (Fig. 10B). Furthermore, the flow cytometry result showed that ROS levels were upregulated in the OGD group compared with the normal group at 72 h exposed to OGD injury (*P* < 0.05). In contrast, the ROS levels were lower in the OGD + TAT-PEP group compared with that in the OGD group at 72 h exposed to OGD injury (*P* < 0.05) (Fig. 10C).

### TAT-PEP Alleviated Neuronal Apoptosis Exposed to OGD Injury

**Fig. 11:**
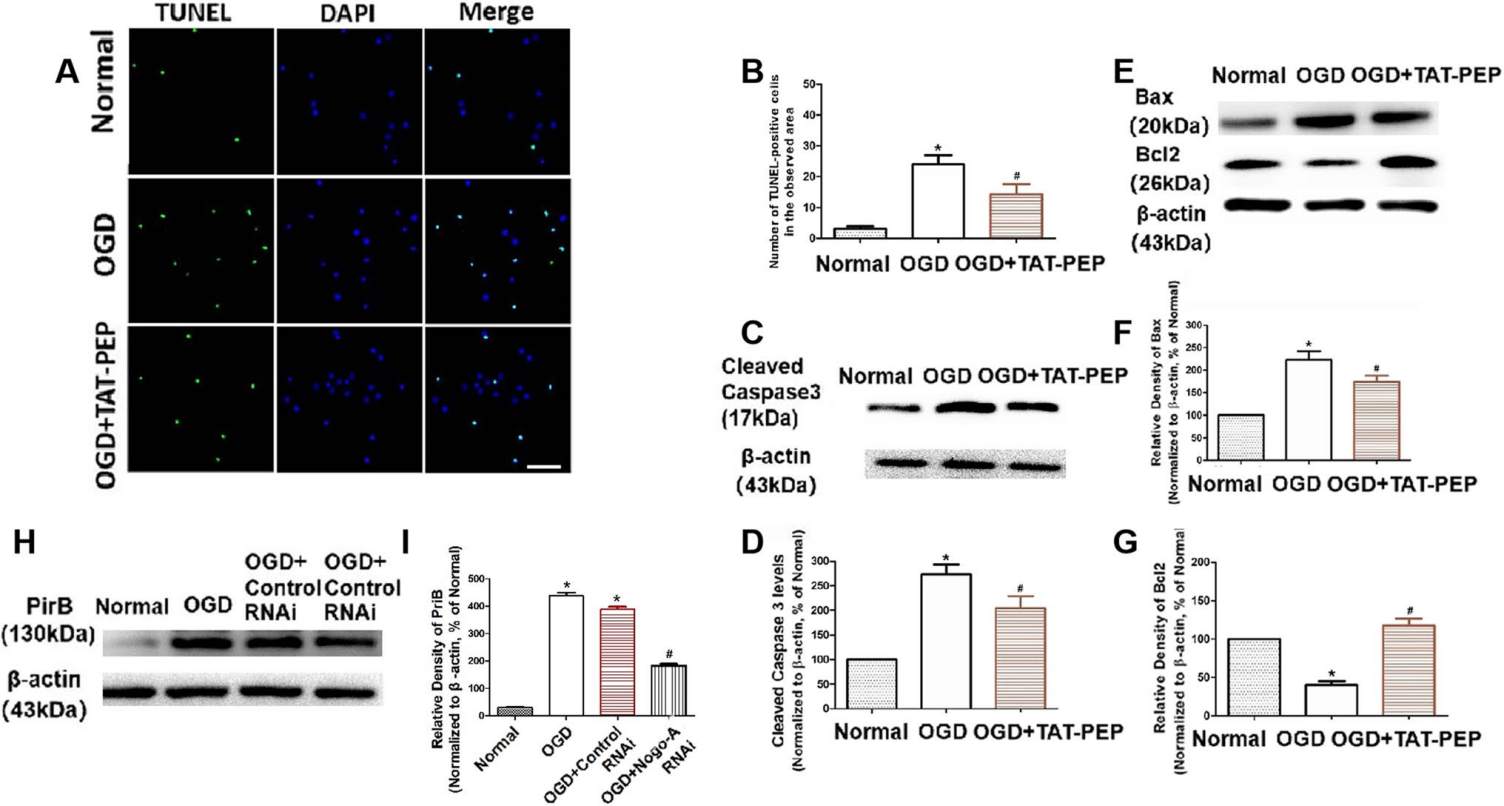
The effect of TAT-PEP on neuronal apoptosis exposed to OGD injury (*n* = 6, each group). **A** TUNEL staining detected the impact of TAT-PEP on neuronal apoptosis at 72 h after OGD. The green cells were TUNEL-positive neurons. Blue represents DAPI-stained nuclei. Bar = 100 μm. **B** It was the statistical diagram of TUNEL positive neurons in each group (*P < 0.05 vs. normal group; #*P* < 0.05 vs. OGD group). **C**, **E** The protein band of cleared caspase, Bax and Bcl2 expression in every group at 72 h after OGD was detected by western blot. **D**, **F**, **G** The statistical diagrams of cleared caspase, Bax and Bcl2 expression in every group at 72 h after OGD. **H** The protein band of PriB expression was detected in every group at 72 h after OGD by western blot. **I** The statistical diagrams of PriB expression after OGD. (*P < 0.05 vs. normal group; #*P* < 0.05 vs. OGD group)

Then, assaying for neuronal apoptosis at 72 h after exposure to OGD, the number of TUNEL-positive cells in the OGD group was more than that in the normal group (*P* < 0.05). In addition, the number of TUNEL-positive cells in the OGD + TAT-PEP group was fewer than in the OGD group (*P* < 0.05) (Fig. 11A, B). The expression of cleaved (active) caspase3, Bcl-2 and Bax was assessed by western blot at 72 h after OGD. Caspase3 activity was significantly inhibited in the OGD + TAT-PEP group compared with the OGD group (*P* < 0.05). Treatment with TAT-PEP increased the level of Bcl-2 protein, whereas it decreased Bax expression compared with the OGD group (*P* < 0.05) (Fig. 11C–G). The expression of PirB was significantly inhibited in the OGD + Nogo-A RNAi group compared with the OGD group (*P* < 0.05). (Fig. 11H, I).

## Discussion

Although advances in prevention and treatment technologies for neurological conditions such as acute stroke, traumatic brain injury and perioperative brain injury have controlled mortality to a certain extent, about 60–80% of survivors will have varying degrees of neurological dysfunction such as cognitive impairment, sensory impairment, motor impairment and communication impairment, which will seriously affect the patients’ quality of life and cause a heavy burden to their families [1, 20]. In particular, ischaemic stroke often involves complex pathophysiological processes that lead to long-term cognitive impairment. This neurological dysfunction is due to damage to the structure and function of the cerebral cortex caused by ischaemia–reperfusion injury. Neurons are the basic unit of central nervous system structure and function. Neurons are susceptible to ischaemia and hypoxia and are prone to degeneration, necrosis and apoptosis, as confirmed in previous experiments [17, 21]. Since Astrup defined the reversible damage beyond the central necrotic area of focal cerebral ischemia as the ischemic penumbra (IP) in 1981, IP has been used clinically as an area for the treatment of ischemic cerebrovascular disease [22]. This is because the IP area slowly progresses to irreversible damage over several hours or 7 days, and neuronal death is mainly apoptosis [12]. Therefore, reducing penumbra neuronal damage and promoting neuronal survival is key to effectively improving neural function after cerebral ischemia–reperfusion injury.

PirB is a co-receptor of Nogo-A, MAG and OMgp, which plays an essential role in axon growth, synaptic plasticity and neuronal survival [5]. In the previous study, we found that the mRNA and protein levels of PirB in the mouse model of cerebral ischemia–reperfusion injury were highly expressed in the ischemic injury area, suggesting that it may play an important role in the pathological process of cerebral ischemia–reperfusion injury [9]. This persistent and significant overexpression phenomenon means that PirB may be a potentially important target for treating cerebral ischemia–reperfusion injury. In the previous study, we first designed and synthesized constructed the transactivator of transcription-PirB extracellular peptide (TAT-PEP), which blocked the interaction between PirB and its ligands and abolished the inhibitory activity of Nogo-A, MAG and OMgp on axonal regeneration. Moreover, TAT-PEP treatment enhanced axonal regeneration and CST projection and improved motor functional recovery after stroke [9]. In the present study, our work also showed that TAT-PEP reversed cognitive dysfunction after ischemic stroke in the Y-maze and novel object recognition tests. The neuroprotective effect of TAT-PEP also suggests that PirB plays an important role in the negative regulation of cognitive function after ischemic stroke. It is also consistent with recent evidence implicating PirB in diabetes-related cognitive impairment through modulation of axonal growth and dendritic remodelling and may represent a potential therapeutic target for cognitive impairment [23]. Another study also showed that PirB is associated with ageing. TAT-PEP may be a promising therapeutic agent for modulating age-related motor and cognitive dysfunction [24]. These results suggest that TAT-PEP could alleviate PirB-associated cognitive dysfunction. This is similar to our findings. However, there may be other mechanisms that need to be investigated.

It remains to be shown how TAT-PEP improves cognitive impairment after ischemic stroke. This study found that TAT-PEP could significantly reduce the volume of cerebral infarction at 3 days after MCAO. In particular, the degree of cortical damage was significantly reduced. Adelson found that the cerebral infarction volume of *pirb* KO mice was significantly reduced 7 days after MCAO compared with *pirb* WT mice [25], which confirmed the above results. Our striking finding then showed that TAT-PEP significantly reduced the number of degenerated and apoptotic neurons in the cortical penumbra, improved neuronal ultrastructure and effectively promoted neuronal survival. This may be an actual reason for TAT-PEP to effectively reduce the volume of cerebral infarction and promote the recovery of cognitive function. In the previous study, using the global cerebral ischemia–reperfusion injury model, our group confirmed that inhibiting PirB function significantly reduced neuronal apoptosis and promoted neuronal survival in the ischemia hippocampus [8]. Clearly, PirB played a vital role in the process of neuronal damage after cerebral ischemia–reperfusion. The above results have further confirmed that PirB may be an important reason for the impairment of neuronal survival and functional recovery after cerebral ischaemia. It is a crucial target for treating ischemic stroke.

As mentioned above, we found that TAT-PEP can play a neuroprotective role, which may be related to its ability to reduce the degeneration and apoptosis of neurons in the cerebral ischemic injury area. Our previous study found that PirB overexpression exacerbated neuronal apoptosis by oxidative stress and mitochondria damage [10]. To further clarify the neuroprotection and mechanism of TAT-PEP, we conducted primary neuron culture and used the OGD model in vitro. The results showed that interfering with neuronal PirB expression or TAT-PEP significantly enhanced the activity of neurons after OGD, reduced the release of LDH and alleviated neuronal damage. Furthermore, TAT-PEP decreased MDA levels, increased SOD activity and helped ROS accumulation of neurons exposed to OGD injury. These results proved PirB participated in neuronal apoptosis caused by oxidative stress after oxygen and glucose deprivation. Inhibiting the function of PirB used, TAT-PEP alleviated oxidative stress and neuronal damage. Although the effects of TAT-PEP on neuronal oxidative stress and apoptosis are still controversial, in our study, the results indicated TAT-PEP decreased MDA level and increased SOD activity, which inhibited lipid peroxidation. TAT-PEP also reduced ROS accumulation which has been suggested to be required for neuronal apoptosis. Lipid peroxidation and ROS accumulation are closely related to the damage of plasma membrane structure in cells. Especially the production of a large number of oxygen free radicals and the lipid peroxidation of a mitochondrial membrane can cause mitochondrial damage and lead to apoptosis. In our vivo study, the ultrastructural changes of mitochondria also confirmed this point. In conclusion, our results provide evidence for the potent neuroprotective effects of TAT-PEP against neuronal oxidative stress, mitochondrial damage and apoptosis after ischaemic stroke.

To further explore the relevant mechanisms, we found that TAT-PEP reduced the number of apoptotic neurons after OGD, inhibited the expression of apoptotic protein Bax, promoted the expression of anti-apoptotic protein Bcl2 and inhibited the activation of Caspase3. Previous studies have shown that the damage of neurons in the penumbra of cerebral ischemia is mainly regulated by apoptosis [26]. One of the mechanisms of neuronal apoptosis in ischemic penumbra is due to mitochondrial damage related to lipid peroxidation and ROS accumulation [27]. It has been reported Caspase-3 is known that the pro-apoptotic protein Bax and the anti-apoptotic protein Bcl-2 can migrate from the cytoplasm to mitochondria, which are distributed in a manner that is consistent with the mitochondrial release of cytochrome C and caspase [21, 28]. The mitochondrial apoptotic pathway plays an essential role in neuronal injury [29, 30], and TAT-PEP inhibited neuronal apoptosis after ischemic stroke by upregulating Bcl-2 expression and reducing Bax expression. The apoptotic proteins on the mitochondrial membrane, such as Bad and Bax, lead to the opening of the mitochondrial permeability transition pore and the release of cytochrome C or apoptosis factors. The activated Caspase3 cleaves DNA repair enzymes, thus leading to DNA damage and apoptosis [31]. However, how PirB regulates the expression of Bax and Bcl-2 after cerebral ischemic-reperfusion injury has not been directly elucidated in this study. We observed the ultrastructure of neurons. We found that the nucleus and mitochondria of neurons in the penumbra were significantly damaged after cerebral ischemia–reperfusion injury. PirB may affect the expression of Bax and Bcl-2, leading to opening of the mitochondrial permeability transition pore and activation of caspase3. However, the specific mechanism and signalling pathway still need to be confirmed. Studies have shown that interfering with the binding of NgR1 and Nogo-A can effectively inhibit the expression of Bax, promote the expression of Bcl-2 and thus inhibit the activation of Caspase3 [32]. However, whether TAT-PEP affects the Nogo-A and PirB pathways, affecting the expression of Bax, Bcl-2 and activated caspase-3, and plays a neuroprotective role, remains to be investigated.

It is noteworthy that a previous study reported that the increased PirB labelling was localized to astrocytes and neurons in lipopolysaccharide (LPS)-treated animals. The hippocampus-dependent spatial learning and memory were impaired [7]. Another study also showed PirB was highly expressed in neurons and astrocytes in the model of epilepsy [33]. It has been reported that astrocytes are mainly responsible for inflammatory injury and cognitive impairment of Alzheimer’s disease (AD) [34]. However, our study did not investigate the expression of PirB in astrocytes and the effect of TAT-PEP on astrocytes after ischemic stroke, which remains to be investigated in future research.

In conclusion, our study showed that TAT-PEP improved neurobehavioral and cognitive function by reducing brain infarct volume and attenuated neuronal degeneration, apoptosis and mitochondrial damage in MCAO. An in vitro study showed that TAT-PEP increased neuronal survival and attenuated apoptosis by reducing MDA levels, increasing SOD activity and reducing ROS accumulation. In addition, the results showed that TAT-PEP alleviated neuronal apoptosis by affecting the expression of apoptosis-associated proteins such as cleaved caspase-3, Bcl-2 and Bax. Taken together, our results suggest that TAT-PEP is a potent neuroprotectant with therapeutic potential in ischemic stroke. In particular, this study suggests that antagonism of PirB function may define an attractive therapeutic strategy against neuronal apoptosis after ischemic stroke in future studies.

## Data Availability

The datasets used and/or analysed during the current study are available from the corresponding author on reasonable request.

## Abbreviations

PirB: Paired immunoglobulin-like receptor B
MAIs: Myelin-associated inhibitory proteins
TAT-PEP: Transcription-PirB extracellular peptide
OGD: Oxygen-glucose deprivation
LDH: Lactate dehydrogenase
MDA: Malondialdehyde
SOD: Superoxide dismutase
ROS: Reactive oxygen species
MAIs: Myelin-associated inhibitory proteins
GP: Glycoprotein
MAG: Myelin-associated glycoprotein
MCAO: Middle cerebral artery occlusion
NOR: Novel object recognition test
MRI: Magnetic resonance imaging
T2WI: T2-weighted images

## Code Availability

GraphPad Prism Version 7.0; ImageJ Version 1.51 J; Imaris Version 9.7.
